# I NEED HELP: AN EXPERIMENT IN COMMUNITY OUTREACH

**DOI:** 10.1093/geroni/igae098.0726

**Published:** 2024-12-31

**Authors:** Jacqueline Angel

**Affiliations:** The University of Texas at Austin, Austin, Texas, United States

## Abstract

This paper describes community outreach activities by faculty affiliated with The UT Austin Texas Aging & Longevity Consortium. Communities across the country like Austin are aging rapidly, while they are becoming more racially diverse and facing an ever-growing need for assistance to older citizens. The City of Austin is a vibrant and cosmopolitan metropolis comprising multiple communities of color—African American, Latino, and Asian which require affordable and culturally-appropriate services and supports. Often these are difficult to find or are simply not available. Many older adults suffer from multiple chronic conditions which increases their need for assistance. As a consequence, community outreach is desperately needed, and investigations in to the most cost-effective and efficient ways of informing clients about available services from which they might benefit is vital. This presentation lays out proposed solutions to confronting these challenges. Specifically, the presentation highlights one award-winning community-based policy research initiative that investigates important possibilities for building an age-friendly Austin that might be applied elsewhere. The public-private initiative uses a multimethod approach to establish an intergenerational community-based resource and activity center (IDC) to optimize space in public places. The IDC model would expand current public health services, including child and adult day care aging, service coordination, and tele-behavioral health in one location. We end with a discussion of lessons learned regarding research-based strategies that encompass three domains: community outreach, co-production, and building partnerships inside and outside the UT campus.

